# fMRI resting state networks and their association with cognitive fluctuations in dementia with Lewy bodies

**DOI:** 10.1016/j.nicl.2014.03.013

**Published:** 2014-03-28

**Authors:** Luis R. Peraza, Marcus Kaiser, Michael Firbank, Sara Graziadio, Laura Bonanni, Marco Onofrj, Sean J. Colloby, Andrew Blamire, John O'Brien, John-Paul Taylor

**Affiliations:** a Institute for Ageing and Health, Newcastle University, Campus for Ageing and Vitality, Newcastle upon Tyne NE4 5PL, UK; b School of Computing Science, Newcastle University, Newcastle upon Tyne NE4 5PL, UK; c Institute of Neuroscience, Newcastle University, Newcastle upon Tyne NE2 4HH, UK; d Department of Brain and Cognitive Sciences, Seoul National University, Seoul, Republic of Korea; e Institute for Ageing and Health, Newcastle University, Wolfson Research Centre, Campus for Ageing and Vitality, Newcastle upon Tyne NE4 5PL, UK; f Department of Neuroscience and Imaging, “G. d'Annunzio” University, Chieti, Italy; g Aging Research Centre, Ce. S.I., “G. d'Annunzio” University Foundation, Chieti, Italy; h Department of Psychiatry, University of Cambridge, Addenbrooke's Hospital, Cambridge CB2 0QC, UK; i Newcastle Magnetic Resonance Centre, Campus for Ageing and Vitality, Newcastle upon Tyne NE4 5PL, UK

**Keywords:** Cognitive fluctuations, Visual hallucinations, Resting state network, Lewy bodies, Dementia

## Abstract

Cognitive fluctuations are a core symptom in dementia with Lewy bodies (DLB) and may relate to pathological alterations in distributed brain networks. To test this we analysed resting state fMRI changes in a cohort of fluctuating DLB patients (*n* = 16) compared with age matched controls (*n* = 17) with the aim of finding functional connectivity (FC) differences between these two groups and whether these associate with cognitive fluctuations in DLB. Resting state networks (RSNs) were estimated using independent component analysis and FC between the RSN maps and the entirety of the brain was assessed using dual regression. The default mode network (DMN) appeared unaffected in DLB compared to controls but significant cluster differences between DLB and controls were found for the left fronto-parietal, temporal, and sensory–motor networks. Desynchronization of a number of cortical and subcortical areas related to the left fronto-parietal network was associated with the severity and frequency of cognitive fluctuations. Our findings provide empirical evidence for the potential role of attention–executive networks in the aetiology of this core symptom in DLB.

## Highlights

• We report resting state network (RSN) alterations in dementia with Lewy bodies (DLB).
• The default mode network was intact in DLB compared to healthy controls (HC).
• Fronto-parietal, temporal, and sensory–motor RSNs showed differences (DLB < HC).
• The left fronto-parietal network (FPN) correlated with cognitive fluctuations in DLB.
• The FPN therefore may be a potential marker for cognitive fluctuations in DLB.

## 1 Introduction

Dementia with Lewy bodies (DLB) accounts for approximately 4–8% of dementia cases (McKeith et al., 2007). It is characterized by complex visual hallucinations (VHs), cognitive fluctuations, and parkinsonism. These three core features help differentiate DLB from other dementias such as Alzheimer's disease (AD) with the presence of at least two out of the three required to make a diagnosis of probable DLB (McKeith et al., 2005). Out of the three core features, probably the least understood is that of cognitive fluctuations and this lack of knowledge has hampered the development of appropriate treatment for this deleterious symptom in DLB (Boström et al., 2007).

As a core feature, cognitive fluctuations may be more specific to DLB than parkinsonism (Tiraboschi et al., 2006). Quantitatively and qualitatively, cognitive fluctuations appear to relate to intrinsic brain processes rather than environmental or situational factors (Bradshaw et al., 2004; Walker et al., 2000), may associate with attentional impairments, and often co-occur with visual hallucinations. Their presence can have significant functional impacts upon patients and their carers (Ballard et al., 2001a; Ballard et al., 2001b).

Neurobiologically, cognitive fluctuations are likely to arise from distributed functional network perturbations rather than specific structural abnormalities (Taylor et al., 2013); on electroencephalography (EEG), increased and fluctuating slow wave activity occurs in posterior regions in DLB patients compared to Alzheimer's disease (AD) patients and these changes have been correlated with the frequency and severity of clinically observed cognitive fluctuations (Bonanni et al., 2008; Walker et al., 2000). Similarly, relative decreases in cerebral perfusion in posterior parietal areas covariant with relative increases in perfusion in distributed motor networks have been observed in fluctuating DLB patients (Taylor et al., 2013). Another approach which may be sensitive to cortical network disturbances associated with cognitive fluctuations is resting state blood oxygen level dependent (BOLD) functional magnetic resonance imaging (fMRI) as this allows the characterization of resting state networks (RSNs) that are task free and thus are not confounded by task dependent differences in cognitive or motor function which may be compromised in patients with dementia and/or parkinsonism.

Current research on RSNs and dementia has focussed mainly on AD, where the current consensus points to a disconnection of the default mode network (DMN) as intrinsic to this type of dementia (see for instance Greicius et al., 2004; Binnewijzend et al., 2012, and Mevel et al., 2011). This network is highly related to consciousness and memory (Andrews-Hanna et al., 2010; Raichle et al., 2001) which are primarily affected in AD. In DLB, recent work by Galvin et al. (2011),Kenny et al. (2012) and Franciotti et al. (2013) has examined the DMN although findings on how it is altered in DLB have been inconsistent which may, in part, relate to methodological and cohort differences between studies.

Given the intimated role of the DMN in internal mentation and its role in attentional/ behavioural performance (Wen et al., 2013) it has been speculated that alterations in the DMN may relate to cognitive fluctuations in DLB. However resting fMRI data on this is limited. A report by Franciotti et al. (2013) focussed on the role of the DMN in DLB cognitive fluctuations and they found, contrary to expectation, that the DMN in DLB patients with cognitive fluctuations was as active as in healthy controls, in contrast to AD patients where it was under-active. It was suggested that this either represented a compensatory attempt to maintain DMN function (Kenny et al., 2012), due to the fact that there is relatively greater pathological load in AD compared to DLB, or that there is a loss of frontal inhibition of the DMN in DLB.

Alternatively, it may be that there is a failure to switch out of the DMN to task positive or attentional networks which is more relevant for attentional lapses (Weissman et al., 2006); Sauer et al. (2006) observed that despite being relatively intact in DLB, the DMN failed to deactivate during motion and colour tasks, which may be indicative of impairments in changing from resting state to focussed attention.

Therefore, RSNs other than the DMN may be more apposite to DLB and the manifestation of cognitive fluctuations, in particular the fronto-parietal networks (also known as executive control networks), and which include both the dorsal attention network (DAN) and ventral attention network (VAN) (Agosta et al., 2012; Beckmann et al., 2005; Fox et al., 2006).

Our questions were therefore to, firstly, establish if the DMN was functionally impaired in DLB patients compared to similarly aged controls and, secondly, in an exploratory data-driven manner, determine what other RSNs aside from the DMN are altered in DLB and if these RSN changes were associated with the severity and frequency of cognitive fluctuations.

In this study we employed a “dual-regression” analysis (Filippini et al., 2009) approach on DLB patients with cognitive fluctuations compared to age matched controls. Dual regression has been used successfully in other studies investigating dementia (e.g. Binnewijzend et al., 2012). In dual regression the selected RSN maps are used in a spatial regression per subject to obtain a single time series which then is regressed again (hence the name of dual regression) to obtain subject specific spatial correlation maps. Dual regression may be superior to using the original independent component time series as seeds since it recovers more features for an individual subject's correlation map.

## 2 Methods

### 2.1 Subjects

Participants (*n* = 16 DLB and *n* = 17 controls) were recruited from the local dwelling population of patients who had been referred to local old age psychiatry and neurology services. Approval for the current study was granted by the Newcastle Ethics Committee.

Diagnosis of DLB was performed by two experienced clinicians using standardized clinical diagnostic criteria. Nine out of the 16 DLB participants had previously undergone dopaminergic imaging and of these all had reduced bilateral uptake of tracer within their striata. Clinical assessments included the Cambridge Cognitive Examination (CAMCOG), Mini-Mental State Examination (MMSE), Neuropsychiatric Inventory (NPI) (Cummings et al., 1994), and the Unified Parkinson's Disease Rating Scale (UPDRS) (Fahn and Elton, 1987). Prior to MRI acquisition, the Clinical Assessment of Fluctuations (CAF) (Walker et al., 2000) was administered to patients to assess cognitive fluctuations; this measure provides a quantification of the frequency and duration of fluctuations in patients. For assessment of visual hallucinations, caregivers were asked to complete the hallucinations subscale of the NPI, with specific reference to the occurrence of visual hallucinations in the past month in terms of severity and frequency (NPI^hall^).

Similarly aged controls were selected from friends and spouses of patients and demonstrated no history of psychiatric or neurological brain disease and an MMSE score > 26. From our DLB group, 13 participants were taking cholinesterase inhibitors, 8 l-DOPA based medications, one of the DLB participants was taking a dopamine agonist, two subjects antidepressants, and two low dose benzodiazepines (clonazepam) for suspected REM-sleep behaviour disorder.

### 2.2 Data acquisition

Imaging was performed using a 3 T Philips Intera Achieva scanner. Structural images were acquired with a magnetization prepared rapid gradient echo (MPRAGE) sequence, sagittal acquisition, echo time 4.6 ms, repetition time 8.3 ms, inversion time 1250 ms, flip angle = 8°, SENSE factor = 2, and in-plane field of view 240 × 240 mm with slice thickness 1.0 mm, yielding a voxel size of 1.0 × 1.0 × 1.0 mm. Resting state scans were obtained with a gradient echo echo-planar imaging (GE-EPI) sequence with 25 contiguous axial slices, 128 volumes, anterior–posterior acquisition, in-plane resolution = 2 × 2 mm, slice thickness = 6 mm, repetition time = 3000 ms, echo time = 40 ms, and field of view = 260 × 260 mm. An axial orientation gradient echo T1 weighted image was also acquired to aid in coregistering the resting state to the structural TR 223 ms, TE 2.3 ms, flip angle 80°, slice thickness 4 mm, and pixel size 1.5 × 1.5 mm.

### 2.3 Analysis of MRI and resting state

Data analysis for RSN inference was performed using time concatenated (controls + DLB subjects) MELODIC from the FMRIB's Software Library (FSL version 4.1; http://www.fmrib.ox.ac.uk/fsl). Pre-processing included FSL tool FLIRT motion correction with spatial smoothing FWHM of 6.0 mm, and high pass filter cutoff equivalent to 150 s. Registration to the MNI152 standard brain for both structural and functional MRI was carried out using FSL tool FNIRT (non-linear coregistration with 10 mm warp resolution). The concatenated volumes were decomposed in 42 spatial component maps. Component maps of interests were selected by visual inspection according to previous literature (Agosta et al., 2012; Beckmann et al., 2005; Damoiseaux et al., 2006) and having a concentrated power spectrum below 0.1 Hz. The maps derived are shown in Supplementary Fig. S1 and included the central and lateral visual networks, DMNs I and II, left (L) and right (R) fronto-parietal, sensory–motor, and temporal networks.

Group averages and between subject analyses for group comparisons were performed using dual-regression (available in FSL 4.1). For statistical significance non-parametric permutation was implemented in dual-regression (10,000 permutations), where corrections for age, sex and grey matter (using feat_gm_prepare script available in FSL 4.1) were also included as covariates in the design matrix. Finally, in order to assess positive relations of the dual-regressed time series we implemented contrast masking, i.e. group comparison results were masked by group average maps (voxels that fell within either DLB or control group average maps, thresholded at *p*-value < 0.05, familywise error (FWE) correction for multiple comparison using threshold free cluster enhancement (TFCE)). No statistical correction for multiple RSNs was implemented.

### 2.4 Statistical analysis of clinical measures.

Statistical results in Table 1 including the two-sample *t*-tests were obtained using R (version 2.15.3, psych library). The association of RSNs was tested by Spearman's rank correlation against the CAF scale for cognitive fluctuations. As part of a secondary analysis we also considered RSN alterations associated with other DLB symptoms including the degree of parkinsonism (UPDRS) and the severity and frequency of visual hallucinations (NPI^hall^) in DLB patients. Statistical significance for regression of clinical measures with seeded significant clusters given by dual regression (subjects' normalized *z*-score images) was tested using nonparametric permutations (10,000 permutations) for Spearman's correlations (with correlation equating zero as null hypothesis) and implemented in Python (scipy.stats library version 0.9.0). Only clusters >10 voxels were analysed for correlation with clinical scores.

**Table 1:** Demographic, clinical and cognitive measures.

	DLB (n = 16)	Controls (n = 17)	*p*-Value
M:F (% female)	13:3 (19%)	14:3 (18%)	χ^2^ = 0.0067, *p* = 0.934^a^
Age	76.2 ± 5.7	77.3 ± 4.7	*t*_31_ = 0.415, *p* = 0.524^b^
MMSE	24.2 ± 3.75	29.1 ± 0.83	*t*_31_ = 27.38, *p* < 0.001^b^
UPDRS total	15.94 ± 5.93	1.41 ± 1.87	*t*_31_ = 92.46, *p* < 0.001^b^
CAMCOG total	78.8 ± 11.9	96.4 ± 3.43	*t*_31_ = 33.95, *p* < 0.001^b^
CAF total	3.56 ± 4.35	na	na
NPI total	8.60 ± 5.59^c^	na	na
NPI hallucinations subscale	1.75 ± 1.84	na	na

Values expressed as mean ± 1SD.

Abbreviations: DLB, dementia with Lewy bodies; MMSE, Mini-Mental State Examination; CAMCOG, Cambridge Cognitive Examination; NPI, Neuropsychiatric Inventory; CAF, Clinical Assessment of Fluctuations; UPDRS, Unified Parkinson's Disease Rating Scale; na, not applicable.

a Chi-square test.

b Student's *t*-test — controls and DLB.

c (*n* = 15).

Furthermore, as an alternative method we also run a non-parametric permutation analysis to further assess relations between the core clinical scores and the significant clusters using the FSL general linear model (GLM) tool to create a one-group design matrix (DLB) with the clinical scores as covariates of interest. Significance was assessed using the FSL randomize function. The non-parametric analysis is shown in Supplementary material, Section 2.

## 3 Results

### 3.1 Demographics and clinical measures

Our study included 16 patients diagnosed with DLB and 17 control subjects. Demographic and clinical scores including relevant subscales are shown in Table 1. Both groups, DLB patients and controls, are matched for age (*p*-values = 0.524).

As expected, compared to controls, the DLB group was cognitively impaired, had a variable degree of cognitive fluctuations as measured by the CAF, as well as evidence of parkinsonism and recurrent visual hallucinations with variable frequency and severity.

### 3.2 Resting state networks and dual-regression

A total of 42 component maps were obtained by MELODIC using default FSL parameters for data dimension estimate (17 component maps were identified as noise or artefactual origin, 11 components were identified as resting state networks, and the remaining 14 maps were of unknown origin). From the identified RSNs, the L/R fronto-parietal, sensory–motor, DMN (I and II), temporal, and medial and lateral visual networks were selected for dual regression. The automatic MELODIC threshold which fits a mixture model to the histogram of intensity values (alternative hypothesis test at *p* > 0.5) for each map was used for visual inspection.

For dual regression, significant decreased FC in DLB compared to controls (DLB < controls; *p*-value < 0.05, FWE corrected for multiple comparisons using TFCE) was found for three networks; the L fronto-parietal, temporal, and sensory–motor networks. No statistical differences were found for the DMN (I and II), the R fronto-parietal, and the medial and lateral visual networks. None of the RSNs showed significant increased FC (DLB > controls; *p*-value < 0.05 FWE corrected).

**Fig. 1:**
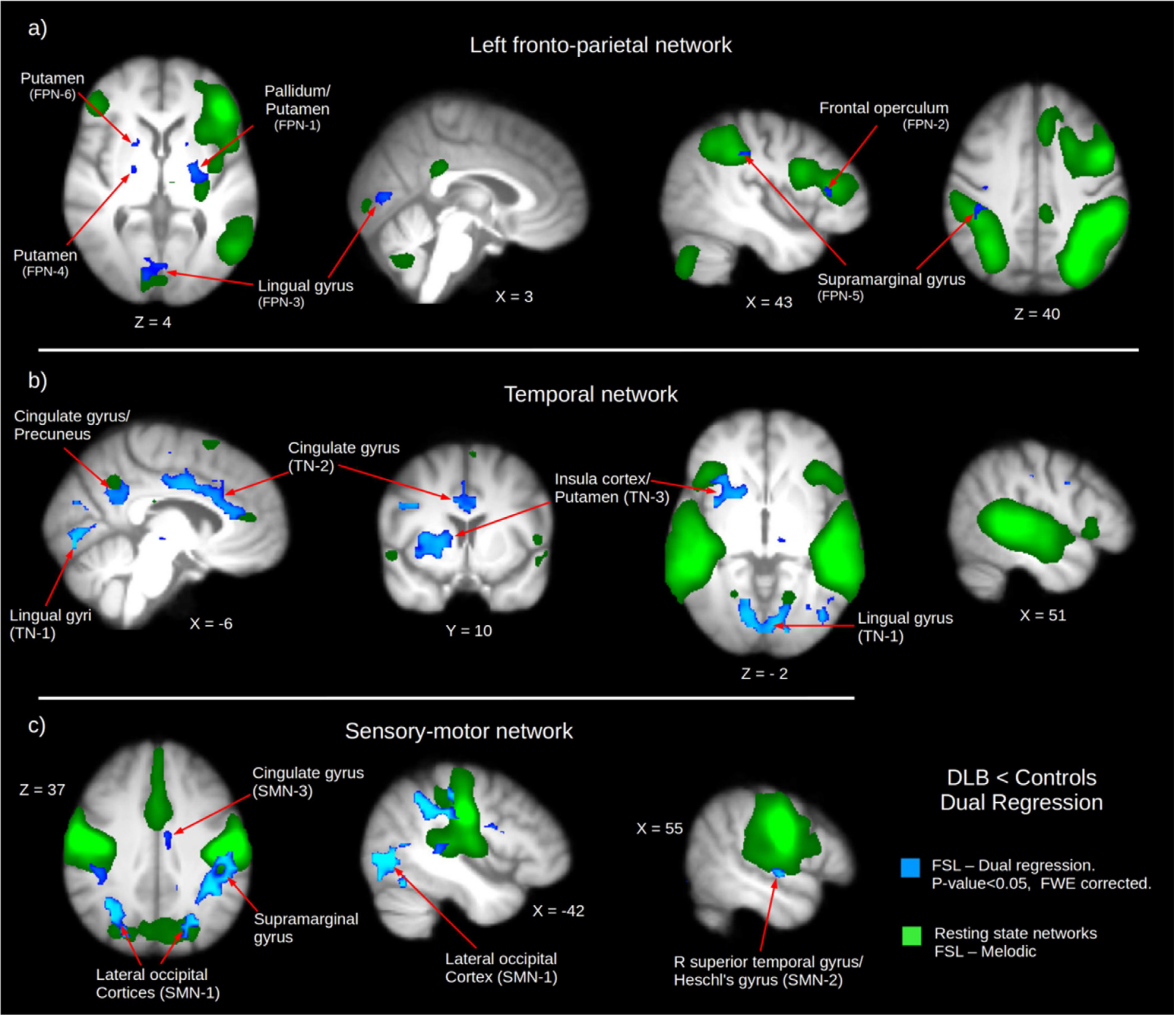
Dual regression significant clusters shown in blue colour (*p*-value < 0.05, FWE corrected). Resting state networks are shown in green. a) Left fronto-parietal network. b) Temporal network. c) Sensory–motor network. Dual-regression results are corrected for sex, age, and grey matter. Brain images are nonlinear coregistered average brains transformed to MNI152 space and shown in radiological convention.

Significant clusters from these networks for the DLB < control comparisons are reported in Table 2. Nine clusters were found for the L fronto-parietal network covering several regions such as the L pallidum, L/R putamen, lingual gyrus, intracalcarine cortices, and R frontal operculum (Fig. 1a). For the temporal network shown in Fig. 1b, fourteen clusters were found covering the L/R lingual gyrus, R putamen, R precentral gyrus, L cingulate gyrus (middle) and L/R intracalcarine cortices. The sensory–motor network showed ten clusters. The largest one widely distributed encompassing both occipital (e.g. L/R lateral occipital cortex, L/R lingual gyrus) and parietal (e.g. L supramarginal gyrus) areas. Two smaller clusters cover the R superior temporal and the L middle cingulate gyri as shown in Fig. 1c.

**Table 2:** Cluster report from dual regression output of significant clusters. All clusters are FWE corrected for multiple comparisons using TFCE. Fronto-parietal network (FPN), sensory–motor network (SMN), temporal network (TN). * indicates the lowest *p*-value region.

	Number of voxels	*p*-Value	MNI (X, Y, Z)	Location
*Cluster code*	*Fronto-parietal network*		[Max *z*-score]	
FPN-1	107	0.022	[−26, −10, 0]	L pallidum*, L putamen
FPN-2	103	0.03	[38, 26, 8]	R frontal operculum*, R inferior frontal gyrus,
FPN-3	93	0.03	[−2, −70, 0]	L lingual gyrus*, L/R intracalcarine cortices, R lingual gyrus
FPN-4	67	0.029	[34, −2, 4]	R putamen*, R pallidum
FPN-5	27	0.033	[34, −30, 32]	R white matter*, R supramarginal gyrus
FPN-6	26	0.027	[14, 10, −4]	R putamen*, R pallidum
	3 clusters < 10 voxels			
				
	*Sensory–motor network*			
SMN-1	2226	0.001	[−42, −74, −4]	L lateral occipital cortex, inferior division*, L/R lingual gyrus, R/L intracalcarine cortex, L/R precentral gyrus, L/R precuneus, L planum temporale
SMN-2	30	0.005	[50, −10, 12]	R superior temporal gyrus, posterior division*, R planum temporale
SMN-3	21	0.034	[−6, −6, 32]	L middle cingulate gyrus*
	7 clusters < 10 voxels			
				
	*Temporal network*			
TN-1	834	0.003	[−10, −82, −12]	L lingual gyrus*, R lingual gyrus, L/R intracalcarine cortex, L lateral occipital cortex, inferior division, L temporal occipital fusiform cortex.
TN-2	786	0.01	[−10, −6, 32]	L cingulate gyrus (middle*), R cingulate gyrus.
TN-3	206	0.01	[38, −2, 4]	R insular cortex*, R putamen, R frontal orbital cortex
TN-4	67	0.011	[34, 6, 24]	R precentral gyrus*, R inferior frontal gyrus
TN-5	21	0.034	[22, −38, −12]	R lingual gyrus*, R parahippocampal gyrus, posterior division
TN-6	15	0.036	[−10, −18, −8]	L white matter*, L brain stem, L thalamus
	8 clusters < 10 voxels

A potential confound affecting the patterning of RSNs, particularly in a neurodegenerative group may have been volumetric loss specific to the DLB group. To test this, we carried out a voxel-based morphometry (VBM) analysis using the statistical parametric mapping software, SPM8 (http://www.fil.ion.ucl.ac.uk/spm/). However we found no structural differences between the groups that may have affected our functional findings (Supplementary material, Section 3); only two significant clusters were identified and these lay external to our functional results given by dual-regression.

### 3.3 Regression analysis with cognitive fluctuations and other clinical variables

Inferred clusters (DLB < controls) were seeded and 15 indices (i.e. those clusters >10 voxels) from the normalized *z*-score images were extracted from the DLB group. Regression results and significant uncorrected *p*-values are shown in Table 3, which shows the clusters where significant correlations were found with the CAF score. Significant correlations for the L fronto-parietal network were found between clusters FPN-1, FPN-3, FPN-4 and FPN-6 (which include the L pallidum, L lingual gyrus, and the R putamen, see Table 2) and the CAF score. Clusters for the temporal and sensory–motor RSNs did not show significant correlations with CAF.

**Table 3:** Correlation with DLB core clinical measures; CAF with dual-regression significant clusters.

Cluster	Spearman's rank correlation (*p*-value)
FPN-1	0.603 (0.0184)
FPN-2	0.482 (0.0630)
FPN-3	0.612 (0.0124)
FPN-4	0.519 (0.0442)
FPN-5	0.066 (0.7926)
FPN-6	0.551 (0.0344)

The non-parametric permutation analysis to assess relations between the CAF scores and LFPN clusters showed similar results (see Supplementary material, Section 2).

## 4 Discussion

In summary, we found from our exploratory dual-regression analysis that the sensory–motor, temporal and L fronto-parietal networks showed significantly lower FC at several regions in DLB patients compared to controls. For the sensory–motor network decreased FC (DLB < controls) was observed in three main clusters covering several regions on posterior areas of the brain (occipital and parietal areas mainly; Fig. 1c); for the temporal network, significantly decreased FC was seen in the L/R lingual gyri and intracalcarine cortices, the L lateral occipital cortex, R insular cortex, the L/R cingulate gyrus, and the temporal occipital fusiform cortex (Fig. 1b). Finally our dual-regression results showed significant clusters for the L fronto-parietal network in regions that include the L/R putamen, L/R pallidum, R frontal operculum, and R supramarginal gyrus (Fig. 1a). In contrast, we were not able to find significant differences in the DMN between DLB patients and the control group and these results concord with previous publications reporting a spared or increased DMN in DLB (Franciotti et al., 2013; Kenny et al., 2012; Sauer et al., 2006). We discuss the implications of these findings apposite to these networks below.

### 4.1 Sensory–motor network

This network is central to the execution of voluntary movements (Biswal et al., 1995) and abnormalities in the functional connectivity of the sensory–motor network have been reported in Parkinson's patients. In addition the topography features of this network may be dopamine dependent (Esposito et al., 2013). Therefore, given the presence of parkinsonism in DLB, it is not unsurprising that we found that FC of this network was affected. Exploratory analysis tentatively supported this as there was a trend association between the severity of parkinsonism as measured by the UPDRS and the functional disconnectivity of this network (Spearman's rank correlation between cluster SMN-1 and UPDRS score: *p*-value = 0.073 uncorrected). The lack of strong relationship may be driven by the tendency to less parkinsonism and known variability in nigrostriatal neuronal loss in DLB (Colloby et al., 2012).

Prior evidence from covariant analyses of perfusion data in DLB have suggested that the expression of both anti-correlated motor (e.g. supplementary motor area and putamen) and non-motor (parietal) networks is intrinsic to cognitive fluctuations and attentional dysfunction in DLB (Taylor et al., 2013). These areas overlap with the sensory–motor network reported here although in the present cohort we found no evidence of an association between CAF and the FC reduction in the sensory–motor network of DLBs. Possible explanations for this may be differences in the sample, investigative modality (resting state vs. perfusion) or analysis approach between the present study and that of Taylor et al. (2013).

### 4.2 Temporal network

This network covers the auditory system, in specific the primary and secondary auditory cortices. Alterations in FC in this network did not associate with cognitive fluctuations in DLB although it is notable that the temporal occipital fusiform cortex is mainly associated with body and face recognition and the lingual gyri on the other hand have been associated with processing of complex images. Certainly visuo-perceptual deficits (Mosimann et al., 2004) and abnormalities in the ventral visual stream (Harding et al., 2002; Taylor et al., 2012) have been reported in DLB. Similarly a diffusion tensor imaging (DTI) study published by Kiuchi et al. (2011) found lower fractional anisotropy (FA) in visual-related areas in DLB patients and lower FA values in bilateral inferior occipitofrontal fasciculus (IOFF; connecting the orbitofrontal cortex with the occipital lobe) and the L inferior longitudinal fasciculus (ILF; connecting the inferior temporal cortex with the occipital lobe). These findings concord with our results showing a disconnection between occipital regions and the temporal RSN. However on our secondary analyses none of the significant clusters related to the temporal RSN correlated with the severity or frequency of visual hallucinations (*p*-values > 0.13) suggesting that FC alterations of the temporal network of DLBs while perhaps being permissive to the manifestation of hallucinations, do not predict, in themselves, hallucination severity or frequency.

Furthermore, we did not see a significant correlation between the thalamic cluster (TN-6) and cognitive fluctuations. This was somewhat surprising given that the thalamus has roles in mediation of arousal and attention (Portas et al., 1998). In DLB, specifically, alterations in thalamic perfusion in DLB patients have also been related to this symptom (O'Brien et al., 2005) and more recent work with functional resting state MRI has also found altered connectivity between the thalamus and frontal and limbic (cingulate cortex) regions (Kenny et al., 2013) although the relationship of this altered connectivity to clinical symptom expression was not described in this paper.

Explanations for the apparent lack of association between thalamic changes in RSN connectivity and cognitive fluctuations in our study may include the lower disease severity of DLB group compared to other studies. However it is notable that thalamic involvement in the manifestation of fluctuations has not been noted in other perfusion studies which take a network perspective (Taylor et al., 2013). Further studies focussing on the structure–function role of the thalamus in DLB which include active attentional task comparisons with resting state may be helpful.

### 4.3 Left fronto-parietal network and default mode network

The fronto-parietal network, also known in the literature as the attentional network, is composed of the VAN and DAN. The VAN is known to respond to task-relevant distractors, and the DAN responds together with the VAN when reorientation of attention is needed (Fox et al., 2006). Even though the attentional system is reported as bilateral for attentional tasks, in resting state it is lateralized for the VAN while the DAN remains bilateral (Fox et al., 2005).

In the present study areas with reduced FC with this network in DLBs included the putamen and pallidum, R frontal operculum, and R supramarginal gyrus; these are areas which have been implicated in the attentional control network (Coull, 2004; Eckert et al., 2009), and specifically we found that the putamen and pallidum bilaterally showed significant correlation with the CAF score. Given the conflation between attention dysfunction and cognitive fluctuations in DLB (Ballard et al., 2001a) it is not unsurprising that attentional networks have implicated in the aetiology of cognitive fluctuations (Bonnani et al., 2008; Franciotti et al., 2013). Interestingly, we did not see any association with FC in this network and the severity of parkinsonism (as measured by the UPDRS) given the association with a number of putamenal clusters. However the fronto-parietal attentional network is not a motor network and thus this finding is perhaps unsurprising; rather the finding of putamen disconnectivity may point towards the cognitive role of subcortical motor areas (van Schouwenburg et al., 2013) and is in tune with previous data implicating motor networks in attentional and cognitive dysfunction in DLB (Taylor et al., 2013).

Our findings of an intact DMN in DLB compared to controls, yet abnormal fronto-parietal network which associates with the CAF, point towards this latter network having a specific role in DLB associated cognitive fluctuations. This is consistent with previous findings presented by Franciotti et al. (2013) suggesting decreased resting state FC between frontal and parietal areas in DLB patients with more marked cognitive fluctuations although this was observed in the right hemisphere rather than the left, unlike the current study.

Lateralization in our results towards the L fronto-parietal network is challenging to explain, although our findings are consistent with previous data by Kiuchi et al. (2011) who observed a lateralization of the DLB pathology towards the left brain hemisphere by a disconnection of white matter tracts.

It is notable that recent work byWymbs et al. (2012) found a relation between both the putamina and the left fronto-parietal network with motor chunking and event segmentation; the latter being a method used by the brain to divide our daily living activities in a set of shorter segments that are concatenated and where attention is increased at the end and start of each event (Kurby and Zacks, 2008) and thus, speculatively, our observation of lower functional connectivity between the left fronto-parietal network with putamenal regions and its correlation with the CAF score might imply that cognitive/attentional fluctuations might relate to aberrant event segmentation although specific task-related paradigms would be needed to test this hypothesis.

### 4.4 Common elements in dysfunctional networks in DLB

All three of the RSNs (L fronto-parietal, temporal, and sensory–motor networks) that displayed reduced FC in DLB compared to controls had functional disconnectivity with occipital lobe structures, specifically the lingual gyrus and calcarine cortices. The ubiquity of desynchronization of the lingual and calcarine gyri that we observed across several RSNs in the present study is in keeping with posterior, occipital changes which occur in this condition (for example, perfusion and metabolism deficits; Lobotesis et al., 2001; Teune et al., 2010; Colloby et al., 2002) and which have been postulated to link with the increased propensity of visuo-perceptual deficits and visual hallucinations which typify DLB (Taylor et al., 2012). However despite evidence of desynchronization of these non-visual RSNs with occipital lobe regions, surprisingly, we did not see any gross differences in functional connectivity in visual RSNs in themselves. This may reflect the variable findings reported across different investigative modalities, on the one hand, demonstrating specific deficits in visual areas in DLB patients (Fong et al., 2011; Sato et al., 2007) and others suggesting, that certainly early/lower visual areas are intact (Taylor et al., 2011; Taylor et al., 2012). The present findings may suggest that abnormalities in the visual system in DLB are arising as a consequence of changes in regions reciprocally connected but external to the visual system and/or in the connectivity of these regions with (e.g. top-down attentional networks) visual areas. For example desynchronization of L fronto-parietal, temporal, and sensory–motor networks with visual areas may be explained by structural connectivity changes in the white matter that connects the occipital lobe to higher association areas and this is supported by a number of DTI studies which have demonstrated occipital white matter abnormalities (Kiuchi et al., 2011; Watson et al., 2012). Alternatively, our failure to see visual RSN abnormalities may be related to the relatively mild cognitive impairment and low visual hallucination symptom score in our cohort (see Table 1); it may be that with more severely affected DLBs, alterations in visual RSNs may become more manifest.

Functional disconnection between controls and DLBs was also evident in the clusters which covered several regions including the putamen and pallidum with regard to both the fronto-parietal and temporal networks. Given the presence of parkinsonism in DLB patients it is not surprising that subcortical motor areas may display abnormalities although we failed to see any clear correlation between these clusters and the severity of parkinsonism on our secondary analyses as measured on the UPDRS. However it may be that these areas are more relevant to cognitive fluctuations since these showed disconnection from the LFPN; in support of this covariant perfusion changes between putamen and parietal areas (Taylor et al., 2013) appear to associate with cognitive fluctuations in DLB (Ballard et al., 2001a), and the present data provide further support for a role in cognitive fluctuations in DLB by subcortical motor networks.

### 4.5 Limitations

The data presented, despite our a priori focus on cognitive fluctuations remains exploratory and thus, in particular, correlations between clinical variables and functional connectivity in DLBs need to be treated with caution and need replication.

Diagnosis of patients was on the basis of antemortem clinical examination rather than pathological diagnosis which represents another potential limitation. However this was mitigated against by the use of standardized clinical criteria and robust, well validated clinical scales and the diagnostic approach applied to the current patient cohort has been shown to have high specificity in autopsy validation studies (McKeith et al., 2000). Another limitation is that 15 out of 16 of the DLB patients were on cholinesterase inhibitors which may have biased our findings; for example Possin et al. (2013) found restored resting state activity compared to healthy controls with improvements in controlled attention in Parkinson's disease patients under rivastigmine treatment. In addition, in our study, the DLB group was relatively mild in terms of cognitive impairment and neuropsychiatric symptoms compared to previous resting state studies in DLB (see for instance Franciotti et al., 2013) and this may contribute to cohort specific differences in resting state findings. However despite our DLB group being cognitively milder and on medications, our patients expressed a wide range of cognitive fluctuations in terms of severity and frequency which strongly coupled with RSN disconnections. Thus we would argue that even in mild DLB, RSN disconnectivity is evident and may be helpful in early diagnosis although our data would need to be contrasted against a control dementia group.

## 5 Conclusions

In conclusion, we found a number of RSNs which were functionally disconnected in DLB compared to controls and specifically that there was an association between disconnectivity of the L fronto-parietal network with cognitive fluctuations. Our results provide support for the concept that cognitive fluctuations in DLB depend upon distributed cortical and subcortical networks and may involve attentional systems. However our present data cannot determine whether disruption to the fronto-parietal RSN is merely correlative with cognitive fluctuations or is actually causally linked.

Further studies characterizing how longitudinal changes in RSNs, mainly the fronto-parietal network, relate to cognitive fluctuations are necessary as well as fMRI attention-task related studies to explore the dynamic switching between default brain states and task-positive networks in DLB.

## Appendix A Supplementary materials

Supplementary for fMRI resting state networks and their association with cognitive fluctuations in dementia with Lewy bodies.

## Footnotes

Appendix A Supplementary material associated with this article can be found, in the online version, at http://dx.doi.org/10.1016/j.nicl.2014.03.013.
