# Supplementary material for: fMRI resting state networks and their association with cognitive fluctuations in dementia with Lewy bodies
Source: Neuroimage Clin. 2014 Mar 28;4:558–65. doi: 10.1016/j.nicl.2014.03.013 (PMC3984441; doi:10.1016/j.nicl.2014.03.013)
Supplement: Supplementary file 1 — Supplementary for fMRI resting state networks and their association with cognitive fluctuations in dementia with Lewy bodies. [file mmc1.docx]

Supplementary Material

1. Resting state networks analysed in dual-regression (controls vs DLB)


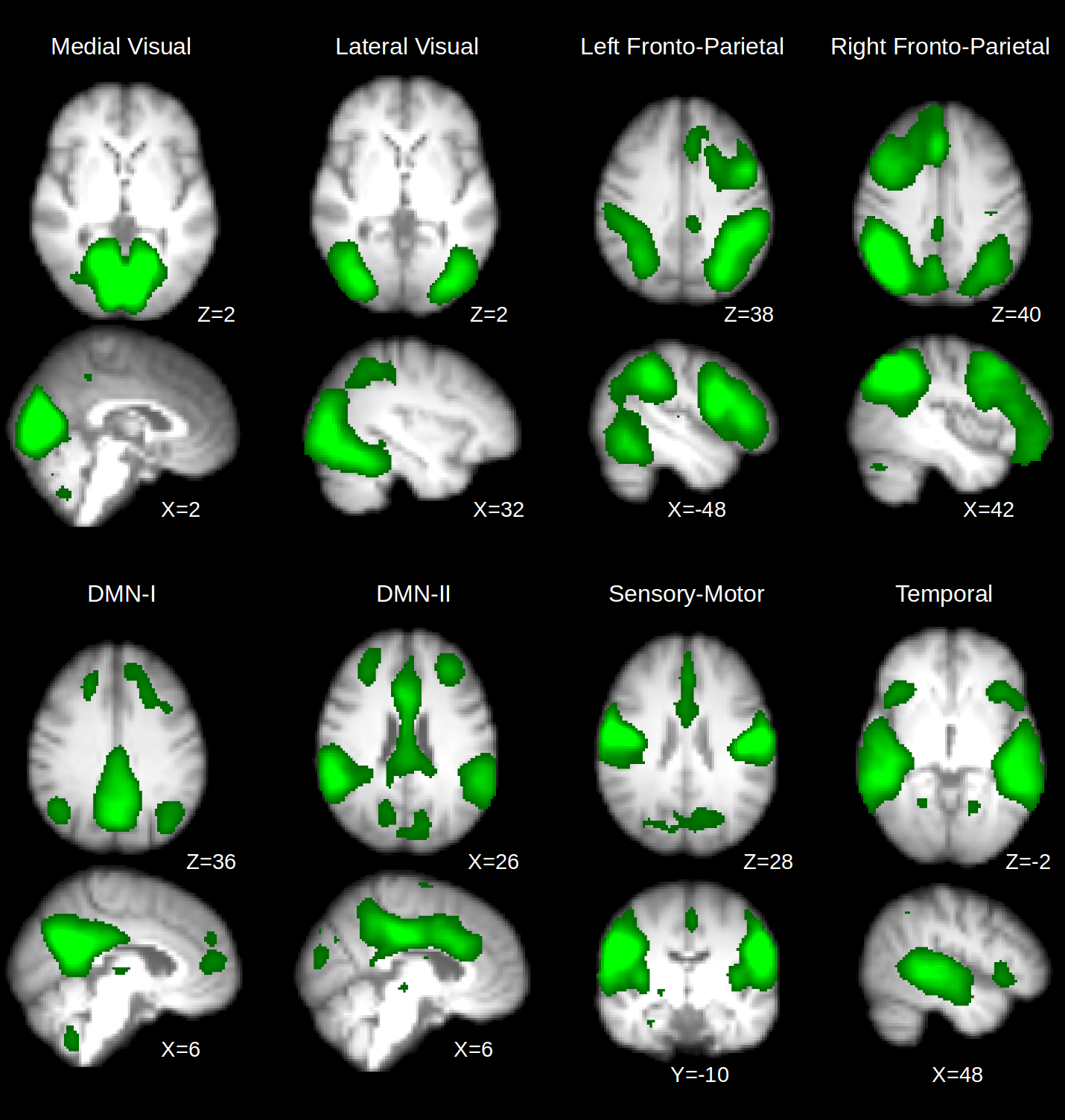


**Figure S1**. A total of eight networks of interest from MELODIC analysis were used for dual-regression. These were (from left to right and from top to bottom) the medial visual network, lateral visual, left fronto-parietal network (LFPN), right fronto-parietal, default mode network (DMN) I, DMN II, sensory-motor network (SMN), and temporal network.

1. Significance between dual-regression clusters from the left fronto-parietal network and CAF clinical scores using a non-parametric approach.

As an alternative analysis to test the robustness of our findings, we did a non-parametric test to find significant relationships between the CAF scores and the dual-regression significant clusters from the left fronto-parietal network. The dual-regression z score volumes for the DLB group were concatenated in a 4D image (16 volumes in total) and the patient CAF scores were used as covariates for the design matrix using the FSL GLM design tool. Statistical significance (uncorrected p-value <0.05) was assessed using randomise (5000 permutations) with a mask for the significant clusters. The mask for the LFPN clusters was composed of 436 voxels and had a total of 9 clusters (see Table II in the main document). Results for this analysis are reported in Supplementary Tables SI and SII (clusters >5 voxels reported)

Since the dual-regression cluster masks were used to limit the randomise analysis to the significant regions, the identified voxels lay within the clusters originally found by dual-regression. For example, a cluster of 37 voxels within cluster FPN-3 (see Table II in the main document and Supplementary Table SI) was the largest one identified by this analysis showing significance between the LFPN and the CAF score.

**Table SI**. Uncorrected p-values for the significant voxels. Significance between the CAF scores and the LFPN DLB clusters.

| CAF score – Left fronto-parietal network significant voxels | | |
| --- | --- | --- |
| Number of significant  Voxels clustered | Localized within dual-regression significant cluster | Max 1-p uncorrected p-value within the cluster |
| 37 | FPN-3 | 0.995 |
| 11 | FPN-1 | 0.970 |
| 8 | FPN-1 | 0.991 |
| 7 | FPN-2 | 0.995 |
| 6 | FPN-2 | 0.972 |
| 10 small clusters < 5 voxels summing 22 voxels | | |
| Total number of uncorrected significant voxels within the mask: 91 voxels | | |
| The mask is composed of 436 voxels. | | |

**Table SII.** Uncorrected p-values for the significant voxels. Significance between the NPI hallucinations score and the LFPN DLB clusters.

| NPI hallucinations score – Left fronto-parietal network significant voxels | | |
| --- | --- | --- |
| Number of significant  Voxels clustered | Localized within dual-regression significant cluster: | Max 1-p uncorrected p-value within the cluster |
| 64 | FPN-3 | 0.998 |
| 6 | FPN-1 | 0.959 |
| 9 small clusters < 5 voxels summing 21 in total | | |
| Total number of uncorrected significant voxels: 91 voxels | | |

The MMSE score showed no significant p-values for the LFPN.

A similar analysis for the sensory-motor network showed only two clusters (of size more than five voxels); one of 58 and another of 11 significant voxels for the NPI hallucinations score. The sensory-motor mask was composed of a total 2307 voxels. CAF and UPDRS did not show significant clusters for this network.

The same analysis for the temporal network and the three clinical scores did not show clusters bigger than or equal to 5 voxels.

In summary, the results relating the CAF score with the LFPN remained regardless of the method, and in fact this relation is the only one that showed to be consistent in both analyses (cluster seeds with Spearman’s rank correlation as presented in the main manuscript and the GLM with non-parametric permutations presented here as supplementary material).

1. Voxel based morphometry (VBM) analysis; DLB vs healthy controls.

To test for structural differences between groups (healthy controls and DLB) a VBM analysis was carried to compare Controls > DLB correcting for age, intracranial volume, and gender. Only two clusters resulted significant by this analysis; the right middle temporal gyrus (posterior division, MNI 66,-22,-6) and the right planum temporale (MNI 59,-31, 18) as shown in Figure S2.


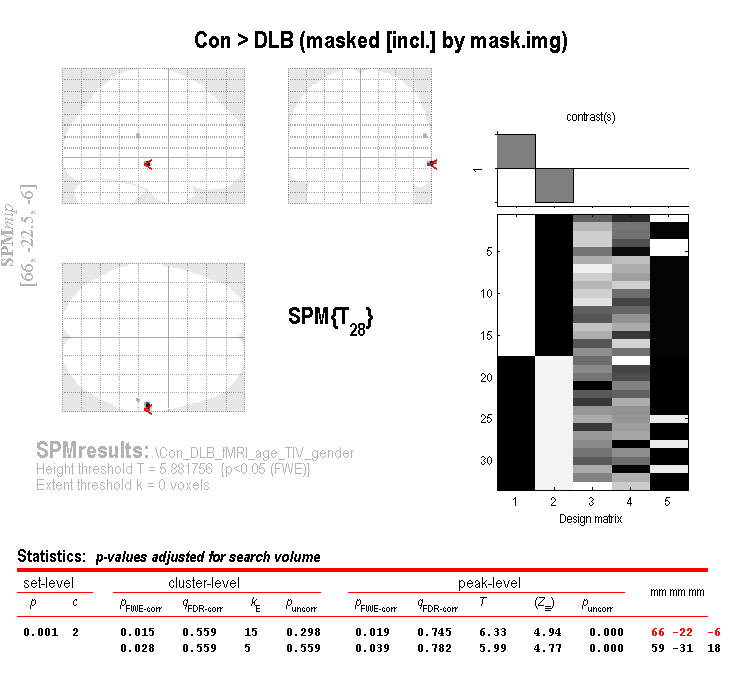


**Figure S2.** Voxel based morphometry results. Only two voxels resulted significant, one located in the right middle temporal gyrus (MNI; 66,-22,-6) and the other in right planum temporale (59,-31,18).

Interestingly, the VBM analysis did not show significant results for the majority of the brain cortex. This is in agreement with previous publications in grey matter analysis reporting lower volumetric losses in DLB compared to AD ([Watson et al., 2012](#_ENREF_1); [Whitwell et al., 2007](#_ENREF_2)) for matched groups. Beyond this our DLB cohort was also relatively mild in terms of their cognitive impairment (MMSE 24.2 ± 3.75) and thus it would not be unreasonable to assume that any volumetric differences with controls might be even less marked than those previously reported. (e.g. Watson et al., 2012; Whitwell et al., 2007).

Watson, R., O'Brien, J.T., Barber, R., Blamire, A.M., 2012. Patterns of gray matter atrophy in dementia with Lewy bodies: a voxel-based morphometry study. Int Psychogeriatr 24, 532-540.

Whitwell, J.L., Weigand, S.D., Shiung, M.M., Boeve, B.F., Ferman, T.J., Smith, G.E., Knopman, D.S., Petersen, R.C., Benarroch, E.E., Josephs, K.A., Jack, C.R., Jr., 2007. Focal atrophy in dementia with Lewy bodies on MRI: a distinct pattern from Alzheimer's disease. Brain 130, 708-719.
